# Supplementary material for: The interactome of intact mitochondria by cross-linking mass spectrometry provides evidence for coexisting respiratory supercomplexes
Source: Mol Cell Proteomics. 2017 Dec 8;17(2):216–32. doi: 10.1074/mcp.RA117.000470 (PMC5795388; doi:10.1074/mcp.RA117.000470)
Supplement: Supplemental Data [file supp_RA117.000470_133922_0_supp_23978_fzffwf.zip › spectra_annotation/mito_DR_spectra_annotation/51-1-8-1-7-1.pdf]

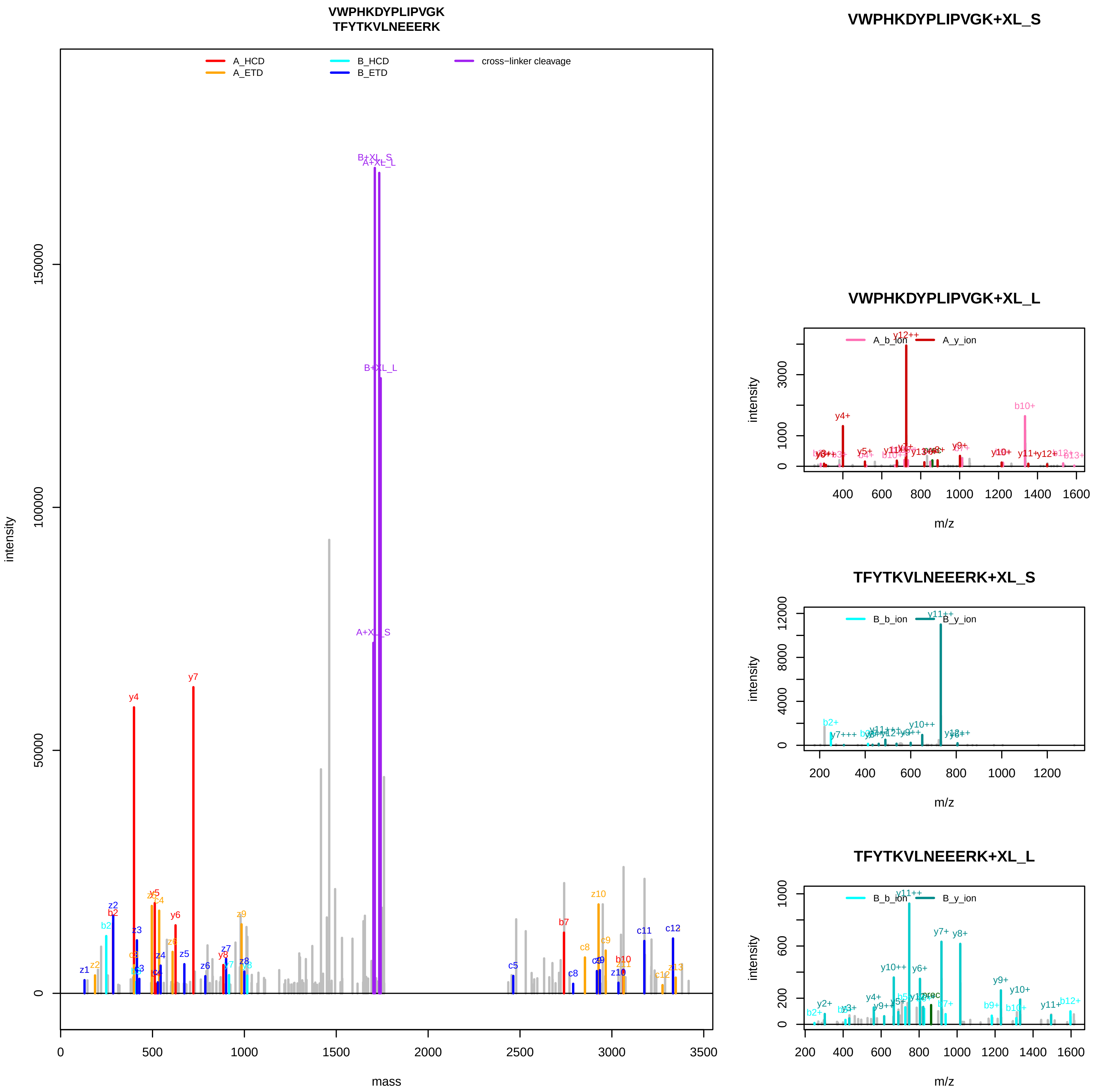
VWPHKDYPLIPVGK
TFYTKVLNEEERK
A_HCD
A_ETD
B_HCD
B_ETD
cross−linker cleavage
B+XL_S
A+XL_L
B+XL_L
A+XL_S
y4
y7
y5
y6
y8
b2
b7
b10
z1
z2
z3
z4
z5
z6
z7
z8
z9
z10
z11
z13
c4
c5
c8
c9
c11
c12
y3
150000
100000
50000
0
intensity
0
500
1000
1500
2000
2500
3000
3500
mass
VWPHKDYPLIPVGK+XL_S
VWPHKDYPLIPVGK+XL_L
A_b_ion
A_y_ion
y12++
y4+
y5+
y9+
y10+
y11+
y12+
b10+
3000
1000
0
400
600
800
1000
1200
1400
1600
m/z
TFYTKVLNEEERK+XL_S
B_b_ion
B_y_ion
y11++
b2+
y7+++
y10++
12000
8000
4000
0
200
400
600
800
1000
1200
m/z
TFYTKVLNEEERK+XL_L
B_b_ion
B_y_ion
y11++
y7+
y8+
y10++
y6+
y9+
y10+
y11+
b12+
y2+
b2+
y4+
1000
600
200
0
200
400
600
800
1000
1200
1400
1600
m/z
intensity
